# *In Vitro* Cultivation of the Hymenoptera Genetic Model, *Nasonia*

**DOI:** 10.1371/journal.pone.0051269

**Published:** 2012-12-05

**Authors:** Robert M. Brucker, Seth R. Bordenstein

**Affiliations:** 1 Department of Biological Sciences, Vanderbilt University, Nashville, Tennessee, United States of America; 2 Department of Pathology, Microbiology, and Immunology Vanderbilt University, Tennessee, United States of America; Oxford Brookes University, United Kingdom

## Abstract

The wasp genus *Nasonia* is a genetic model with unique advantages for the study of interspecific differences, including haplodiploidy and interfertile species. However, as a parasitoid, *Nasonia* is confined within a fly host, thus restricting direct observations and manipulation of development over time. Here, we present the first *in vitro* cultivation method for this system that decouples *Nasonia* from its host, allowing continuous observations from embryo to adulthood. Using transwell plates and a simple *Nasonia* rearing medium, we demonstrate a technique that will significantly expand the utility of the *Nasonia* model.

## Introduction

The genus *Nasonia* is a genetic model for research in evolution, behavior, development, and symbiosis [1]. *Nasonia* is a versatile system with four interfertile species (*N. vitripennis*, *N. giraulti*, *N. longicornis*, and *N. oneida*), three completely sequenced genomes [2], two-week generation times, and simple rearing methods. The haploid genetics afforded by haplodiploid sex determination - in which diploid females develop from fertilized eggs and haploid males develop from unfertilized eggs - is a major advantage for dissecting complex traits, epistasis, and recessive genetic factors [3].

As parasitic wasps, *Nasonia* require an insect host within the Dipteran family (typically *Sarcophaga bullata* flesh flies in the laboratory [4]) to complete its lifecycle. Female *Nasonia* sting the pupa of their Dipteran host to paralyze it, and then oviposit eggs through the fly puparium and directly onto the surface of the developing fly pupa. Eggs hatch and the emerging larvae ectoparasitically feed and develop within the host puparium. This intimate association between the wasp and fly host is a challenge for researchers who focus on biological processes throughout *Nasonia* development because there is a hindrance to studying features of the animal’s lifecycle that are restricted to within-host development and therefore not amenable to continuous observation. Typically, the total time for *Nasonia* development from egg to adult takes 14–17 d at 25**°**C. The first 7–10 d are periods of larval feeding and growth. The remaining time is spent as pupae until eclosure as mature adults within the host fly puparium. Opening the fly puparium before the end of larval development leads to desiccation of the *Nasonia* larvae [5], [6]. As such, it is difficult to conduct continuous, developmental studies of the same individual from embryonic development through the larval instar stages.

**Figure 1:**
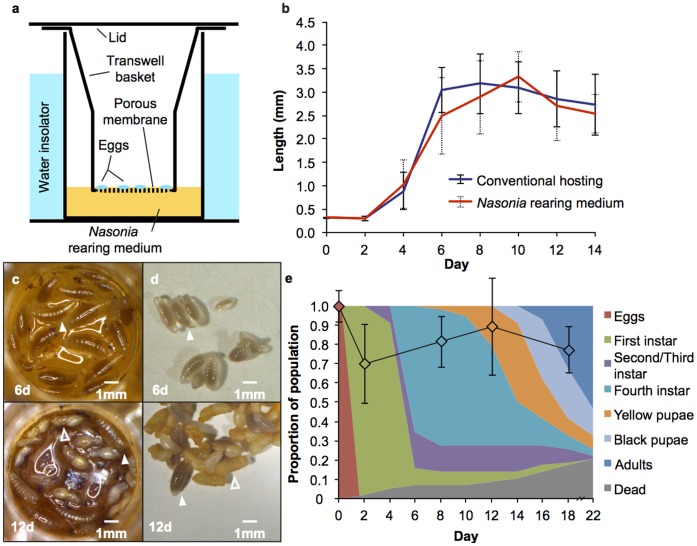
Comparative analysis of conventionally reared *Nasonia* and the artificial rearing assay. a) Schematic of *Nasonia* rearing chamber. Eggs are deposited onto the three micron porous membrane of a transwell basket; the basket is then placed within the well containing 25 µl of *Nasonia* rearing medium so that the two surfaces come in contact with each other, bathing the eggs in nutrients. b) A plot of anterior-posterior measurements during *N. vitripennis* development in conventional rearing conditions (blue line) and in the *Nasonia* rearing medium (red line). Each data point is an average of 150 individuals with standard deviations. c) Photos of *N. vitripennis* larvae in the rearing chamber with *Nasonia* rearing medium 6 d and 12 d post hatching, fourth instar larvae at solid arrow and pupae at outlined arrow. d) Photos of conventionally reared *Nasonia* 6 d and 12 d post hatching. e) Proportional distribution of developmental stages in *N. vitripennis* reared conventionally and on the liquid medium. The *Nasonia* rearing medium allows for direct observation of individuals over time; thus, actual proportions of the population in any given developmental stage are displayed in the stacked area graph, n = 58 individuals. The average number at each stage as a proportion of the average number of eggs laid is depicted by the line graph with standard deviations. The color inside the diamond indicates which developmental stage was counted on a particular day.

To tackle the limitation of the wasp’s lifestyle and strengthen the utility of *Nasonia* as a biological model, we developed a novel technique for rearing *Nasonia* outside of its fly puparium, as well as a method for *in vitro* culturing the fly without its carrion food source. The *Nasonia* rearing technique requires the simple use of transwell plates (Fig. 1a) and a liquid medium containing processed fly pupae (hereafter referred to as *Nasonia* rearing medium). Previous investigations have cultivated parasitoids outside their hosts under a variety of nutrient conditions and as liquid, gel, or encapsulated media environments (as reviewed in [7]). Although there are similar methodologies for rearing parasitoids, with the nutritional composition of the media being the major limiting factor for parasitoid rearing, our rearing method is unique for its use of a transwell rearing chamber, and it is the first *in vitro* cultivation method of the *Nasonia* model.

### Experiments

We demonstrate that *in vitro* cultivation of *N. vitripennis* on the rearing medium yields larval sizes comparable to that of larvae conventionally reared on the fly pupae. Measurements of anterior-posterior length over time indicate that growth rate of the larvae on the *Nasonia* rearing medium is similar to that of larvae from a fly host at the same time point (Figs. 1b–d; t-test for days 0–14, p≥0.158). However, development differs slightly. Specifically, there is a lengthening of the developmental time to reach pupation and adulthood on *Nasonia* rearing medium (Fig. 1e). The most notable difference occurs in the larval stages in which *in vitro* cultivated *Nasonia* experience an extended larval stage 4 instar (p<0.001, t-test). There is also a reduction in number of adults that eclose *in vitro*, from 70.3% emerging in conventional rearing compared to 53.5% in *Nasonia* rearing media (Fig. 1e, p<0.001, t-test). The other two species, *N. giraulti* and *N. longicornis,* were reared successfully on the *Nasonia* rearing medium as well. 69.7% of *N. giraulti* eclose as adults on the rearing medium compared to 74.6% on conventional host rearing (p = 0.957, t-test). Similarly, 61.6% of *N. longicornis* eclose on the rearing medium compared to 74.8% on conventional hosts (p = 0.908, t-test).

The delay in larval development on *Nasonia* rearing medium is due to at least two variables: availability of nutrients early in development and level of humidity within the chamber. The difference in developmental time observed in other *in vitro* cultivated parasitoid species has been associated with restricted nutritional components of the artificial diet [7]. When developing this protocol, we first provided fresh media every third day. Under these conditions, we observed fratricide by *Nasonia* larvae eating their well-mates, and those larvae that did survive never developed past their fourth instar. The fratricidal cannibalism has never previously been observed in *Nasonia* and could be a strategy for the parasitoid in periods of limited resources. Increasing the frequency at which media was replaced from every three days to two days improved development and eliminated fratricide. Also, removing the availability of *Nasonia* rearing medium shortly after the larvae enter their final instar can improve the rate of pupation. However, without an increase in humidity, larvae lose mass and desiccate as early pupae. Thus, keeping humidity high increased successful pupae development.

**Figure 2:**
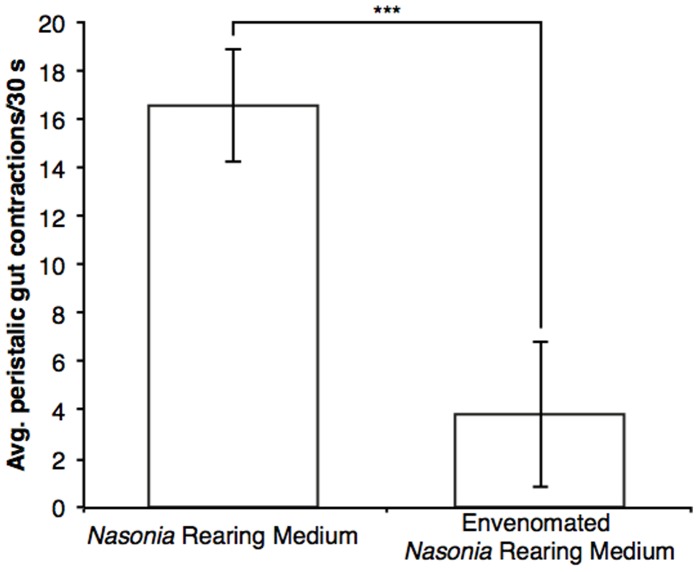
Peristaltic action of larval gut reared on *Nasonia* rearing medium with and without maternal venom. Average rate (±SD) of gut peristalses as observed in first instar larvae (1–2 d post eclosion) for 30 s. For each rearing condition, 35 individual larvae were observed between six replicates (*** = p<0.001, t-test).

When *Nasonia* females first parasitize a host pupa, they inject venom that arrests the development of the fly host. To test whether the utility of the *Nasonia* rearing medium is enhanced by the presence of the venom, the *Nasonia* rearing medium was made from parasitized fly hosts after 8 hr of exposure to virgin female *Nasonia*. Results demonstrated that larval development arrested in all 1^st^ instar larvae after they hatched from their egg casings and began to feed. Peristaltic action of larval gut was observed in these larvae at an average rate of 3.8±3.0 contractions in 30 s (Fig. 2). This rate is more than five-fold slower than the rate of their brothers reared on *Nasonia* medium that lacked the venom, 16.6±2.3 contractions in 30 s (p<0.001, t-test). Ultimately, all larvae were dead by the sixth day of observation. This result indicates that components of the *Nasonia* venom in the rearing media are non-specific and arrest *Nasonia* larval growth and development. Considering that *Nasonia* is an ectoparasitoid, conventionally raised *Nasonia* larvae may have little contact with the components of the maternal venom. However, the *Nasonia* rearing medium itself could alter or concentrate the venom compounds and increase non-specificity of the venom.

### Conclusions

Overall, the ability to chronicle individuals’ development from embryo to adulthood is a feature of this model system that was missing prior to this technique. In conventionally-reared *Nasonia*, obtaining the developmental stages for a given time point would kill the wasps and terminate the samples if they were not already in the pupal stage. This approach required additional replicates of an experiment if one wanted to observe responses in development throughout the *Nasonia* lifecycle, in particular larval development and early pupation.

The *Nasonia* rearing medium untethers the model parasitoid from its host. In conjunction with already established molecular techniques, such as RNA interference [5], [6] to alter specific gene regulation, this technique can expand the range of *Nasonia* investigations. Regular inspections of the wells for larval eclosure, growth, behavior, and development are easily performed without physically disturbing the larvae or exposing them to conditions outside the well. Additionally, muscular kinetics, gut morphology/peristalsis, neurological development, tracheal systems, and fat bodies can be documented due to the translucent tissues of the larvae. Further development of molecular and histological techniques for *Nasonia* will increase the utility of this rearing method and the model system to broader research interests.

## Materials and Methods

### *Nasonia* Rearing Medium and Setup

*Nasonia* rearing medium was made using approximately 55 g of *S. bullata* pupae (approximately 1.5 cm in length) that were submerged for 5 min in a 10% bleach solution. The pupae were then rinsed with sterile millipore water before incubating in a final volume of 100 ml sterile millipore water at 36**°**C for 25 min. The pupae were then homogenized and filtered using a 75 µm nylon mesh sterile cell strainer (Fisherbrand, Fisher Scientific) to remove large, unhomogenized particles. The filtrate was then centrifuged for 5 min at 4**°**C (25,000×G). The centrifuged filtrate separates into three distinct layers, the bottom and top layers consisted of insoluble lipids and exoskeleton debris that were discarded while the middle layer is pipetted into a sterile beaker. Then 50 ml of Schneider’s *Drosophila* Medium 1× with L-Glutamine (GIBCO, Invitrogen) and 10 ml of Fetal Bovine Serum (GIBCO, Invitrogen) were combined with the liquid *S. bullata* (from the middle layer). The mixture was then filtered using a 0.44 µm pore, sterile filter. The flow-through was filtered again using a sterile 0.22 µm pore filter. Finally, 2 ml each of carbenicillin and penicillin/streptamycin (Research Products International Corporation, Mt. Prospects, IL) was added at 100 µg/ml. The completed medium was stored at 4**°**C for up to 2 weeks or frozen at −20**°**C for up to 2 months. Upon thawing, the medium needs to be warmed to 34**°**C and filtered again as particulates will form.

### *Nasonia* Rearing Medium Growth Assays

Each virgin *Nasonia* female was hosted on 2–3 pupae of the fly *S. bullata* for 8 hr. Eggs were then collected using a blunt probe, placed on a Transwell Permeable Support 6.5 mm polyester membrane with a 3.0 µm pore in 24 well plates (Costar, Corning Incorporated, Corning, NY), rinsed with a 10% bleach solution for 1 min, and then rinsed with sterile millipore water. Collecting *Nasonia* eggs for the rearing medium is the most challenging aspect of this assay. Finally, 250 µl of *Nasonia* rearing medium was aliquoted into the well so that the media touches the bottom of each membrane and forms a meniscus around the eggs without completely submerging them. Each well was photographed and the final numbers of eggs were tallied (an average of 18 eggs/well). All transwell plates were stored in a sterile chamber kept at 25**°**C and around 40% humidity. After 10–12 d, the fourth instar larvae on the transwell basket membrane were removed from the media and placed in a clean sterile well, with sterile water in empty wells to maintain high humidity. See references for descriptions on maintenance [8], virgin collection [9] and egg collection [10] of *Nasonia*.

Each well was tallied and/or photographed every other day for 14–22 d throughout the experiments. Anterior-posterior lengths were calculated using Adobe Photoshop CS5.1 analytic tools. Confirmation of living individuals was done visually by confirming either peristaltic contraction of the visible, grey gut or through observation of body movement.

### Conventional *Nasonia* Rearing

Concurrent to the *Nasonia* rearing medium growth assays, sister virgin female *Nasonia* were likewise hosted on fly pupae to observe growth and development compared to their media-reared cousins. For each observation of growth and/or development, a total of 10–15 hosts were opened and individual *Nasonia* were counted (approximately 400 individuals per stage). To estimate the average proportion of conventionally-reared *Nasonia* that develop to a specific developmental stage, a cohort of fly pupae were parasitized on the same day and a subset were selected at random at 0 d (n = 12 hosts) to determine the average number of eggs laid. Different random subsets of hosts were used to calculate the average number of first instar larvae (d 2, 12 hosts), fourth instar larvae (d 8, 10 hosts), yellow pupae (d 12, 10 hosts) and adults (d 18, 10 hosts) that developed. The average proportion of *Nasonia* that survived to each stage was calculated by dividing the average number of *Nasonia* per host at a particular stage by the average number of eggs per host.

### Maternal Venom Experiment

Two containers of fly pupae were weighed out at 60 g each. Virgin *N. vitripennis* females were distributed among one container (2–3 hosts per female) for 8 h at 25**°**C before being removed. The second container was maintained in the same conditions but without being exposed to *Nasonia*. To confirm that the batch exposed to *Nasonia* was parasitized, a random subset of fly pupa was visually inspected for envenomation (black mark on the fly pupal surface). Each batch of hosts was processed into the *Nasonia* rearing medium as described above.

Concurrently, virgin *N. vitripennis* females were hosted on fly pupa for egg collection and set up for growth assays as previously described. Approximately 15 eggs were placed in per transwell across 12 wells containing one of the two media. Each media type (*Nasonia* rearing medium and *Nasonia* rearing medium with venom) was replicated six times.

Rate of the gut peristaltic movement was determined by counting the number of contractions in 30 s. Individuals were chosen at random (four/well) across the six replicates for each experimental treatment. Any dead larvae were excluded from the peristaltic assessment.

### *Nasonia* Strains

In developing the *Nasonia* rearing medium, *N. vitripennis* (strain 13.2), *N. giraulti* (strain RV2x(u)) and *N. longicornis* (strain IV7R3-1b) were used. Growth assays for *N. giraulti* were replicated for a total of 11 times with 6–12 transwells per replication; *N. vitripennis* was replicated eight times with 6–12 transwells per replication; and *N. longicornis* was replicated six times with 6–12 transwells per replication. A control transwell of phosphate saline buffer (pH 7.4) was maintained in each replicated experiment, though the first instar larvae from eggs placed in these wells die within 24–48 hours of eclosing. All strains have been maintained in our laboratory at 25**°**C in constant light.

#### *In vitro* cultivation of *Sarcophaga bullata* flesh flies

All laboratory-reared *Nasonia* strains are traditionally reared on the pupae of *S. bullata*, a fly species that lays its eggs in rotting meat for its larvae to feed upon. These flesh flies are reared in the laboratory using beef liver as an ovipositioning platform and larval food. It takes approximately seven days of larval feeding on the liver before they are mature enough to pupate. The pupae are aged for two to three additional days before being harvested for use in *Nasonia* rearing (see Werren and Loelin 2009, *Cold Spring Harbor Protocols* for additional details). While effective and regularly used by *Nasonia* labs, this method of rearing *S. bullata* does not afford a controlled condition for studying *Nasonia* immunity, development, or microbial symbiosis. The basic reason is that being a carrion feeder, the fly acquires microorganisms from its environment that the *Nasonia* acquire when they feed on the fly (see Brucker and Bordenstein 2011). Here, we report a technique for *in vitro* rearing of *S. bullata* to pupation on a sterile diet.

The *S. bullata* rearing medium is agar based and can be used to successfully induce ovipositioning of adult female flies. Fresh medium was made by adding 18.5 g of BBL™ Brian Heart Infusion (Becton, Dickinson and Company, Spark, MD, USA) and 3.75 g of Agar (Fisher Scientific) to 450 ml of sterile millipore water. After mixture, the solution is autoclaved at 121**°**C for 15 min. After cooling to 50**°**C, 50 ml of room temperature defibrinated sheep’s blood (HemoStat Laboratories) was gently mixed into the suspension. 2 ml each of carbenicillin and penicillin/streptamycin (Research Products International Corporation, Mt. Prospects, IL) was then added at 100 µg/ml; 10 ml of the antifungal Methyl p-Hydroxybenzoate (Fisher Scientific) was also added at 20% concentration by volume in 200 proof ethanol. 12 ml aliquots of the mixture were poured into 90 mm sterile petri dishes (Fisher Scientific) and allowed to cool overnight. Plates can be stored, covered in a plastic bag in the dark at 4**°**C for up to one month.

Before use, the *S. bullata* rearing medium plates are warmed to room temp. Fly eggs and newly eclosed larvae are collected from the liver used in culturing the insect. Additionally, when *in vitro* cultivated *S. bullata* adults were provided without any liver, they willingly oviposited onto the rearing medium plate over an 8 hr period, though qualitative observations indicated fewer overall egg deposits compared to liver. The eggs and larvae are then submerged in a 9∶1 water to household bleach solution for 4 min to remove any transient bacteria or fungi on their surface before transferring onto the medium plate. Approximately 15–30 eggs or larvae are maintained on a single plate. The egg or larval plates are then stored in a sterile lidded container and incubated at 30**°**C. Checked daily, the larvae grew and tended to burrow into the medium. Venting the chamber by regularly opening the container in a UV sterilized hood was important as we noted that ammonia buildup adversely affected the developing larvae. Larvae were transferred to sterile containers with sterile paper towel at the bottom on the eighth day while continuously incubated. Pupation occurred within one to three days and were viable for *Nasonia* rearing. When *N. vitripennis* females were given access to a cohort of 24 *S. bullata* that were reared conventionally, an average of 33±13 viable adults per host emerged. Likewise, when the flies were reared on *S. bullata* rearing medium, a cohort of 24 parasitized fly pupae had an average of 36±15 adult *Nasonia* emerge (p = 0.453, t-test).

Interestingly, when the *S. bullata* were reared on medium with antibiotics, PCR and culturing methods of detecting bacteria (using 16S rDNA primers and culture techniques described in Brucker and Bordenstein. 2011) indicated no detectable levels of bacteria present in the system. However, when medium was tested without the use of antibiotics, the eggs and larvae still contain bacteria and fungus which quickly overgrew the dish, killing the developing larvae. The microbial contamination was mitigated, however, by regularly transferring the developing larvae onto new medium every day, with subsequent rinses in 10% bleach solution between transfers.

## Protocol

### Preparing *Nasonia* Rearing Medium

1. Weigh out 55 g of *S. bullata* hosts into an autoclaved beaker. (Note: Discard any hosts or dead larvae that are not good quality before weighing.)
2. Fill the beaker with household bleach diluted to 10%, by volume, in water, to cover the hosts. Gently agitate the hosts in the solution for 5 min.
3. Pour off the bleach solution and rinse hosts with sterile water to remove the remaining bleach.
4. Add sterile water to the hosts in the beaker to approximately 2/3 the volume of hosts.
5. Cover the beaker and place in a 36**°**C water bath for 25 min.
6. Homogenize the host pupae. (Note: A sterilized and warmed kitchen blender is an efficient means of evenly and rapidly homogenizing the hosts.)
7. Quickly, pass the homogenate through a 75 µm nylon mesh sterile cell strainer into sterile 50 ml conical tubes to remove large particulates.
8. Centrifuge the filtered homogenate for 5 min at 4**°**C (25,000×g).
9. The homogenate will separate into three distinct layers; pipette the middle layer into a sterile beaker and discard the top and bottom layer.
10. Combine the homogenate with 50 ml of Schneider’s *Drosophila* Medium 1× and 20% Fetal Bovine Serum.
11. Filter the mixture with a 0.44 µm pore, sterile filter. (Note: Larger pore sizes can be used to prefilter the mixture, passaging it through smaller pore sizes each time; prefiltering can decrease the total handling time of the mixture.)
12. Filter the flow-through using a sterile 0.22 µm pore filter. The final filtrate should be a translucent honey color. (Note: the *Nasonia* rearing medium is ready for use, however we recommend adding antibiotics to the medium as bacteria can rapidly overgrow and kill *Nasonia* in the assay.)
13. Add 200 µg each of carbenicillin and penicillin/streptamycin. (Note: Alternative antibiotics, chemicals, and concentrations can be supplemented in this step.)
14. Store at 4**°**C for up to 2 weeks or frozen at −20**°**C for up to 2 months. (Note: Upon thawing, the medium needs to be warmed to 34**°**C and filtered again as particulates will form.)

### Setting up *Nasonia* Rearing Medium Growth Assays

1. Working in a sterile laminar flow hood, allow *Nasonia* rearing medium to warm to room temperature.
2. Collect *Nasonia* embryos or larvae to be used in the assay.
3. Place *Nasonia* into the transwell basket(s) and rinse with 10% bleach solution to sterilize their surface for 45 s.
4. Remove bleach solution and rinse *Nasonia* with sterile water, removing excess water when *Nasonia* are fully rinsed. (Note: Prolonged exposure to the bleach solution is harmful to the *Nasonia*, thus it is important to work quickly to rinse away any remaining bleach.)
5. Pipette 250 µl of *Nasonia* rearing medium into the well. The media should contact the bottom of the transwell basket containing the *Nasonia*. (Note: The volume of media needed will vary depending on the size of the well; in this study we used 24 well plates.)
6. Using sterile water, fill space around the wells or in empty neighboring wells to increase humidity.
7. The assay is now ready. Transfer the transwell baskets to new, sterile, wells containing fresh medium every 24–48 hrs based on bacterial contamination or evaporation.
